# 

**Published:** 1858-04

**Authors:** 


### CONTENTS.

Introduction.............................. 1

Excavators, by J. Taft, D. D. S............ 5

Phosphate of Lime Lozenges............ 9

O. & H's. Teeth and Taft's Pluggers...... 10

Blakesley's Method of Filling Teeth...... 11

Cheoplasty............................ 14

*Instructions for Mount'g Teeth by casting 16

Report of the Mississippi Val. Association. 22

American Dental Convention............ 39

Diseased Antrum, by D. W. Roudebush... 39

Rimming Plates by S. Wardell........... 40

Dr. Hoys New Dental Lamp............. 41

Double End Excavators, by Hoy......... 43

Correspondents and Subscribers.....43 & 44

Apology and Advertisers................ 45

Hard Rubber Base by Samuel Mallett..... 46

Extracting Teeth by A. J. Howe........ 50

False Business Practice................. 57

New Dental Lathe....................... 57

Forceps with Shifting Beaks............. 57

Currency Bankable in Cincinnati........ 58

Cincinnati Produce Market.............. 58

Advertisements........................59 60 61 62 63 & 64

* In first line of this article for *exact* read *extract*.

